# Amplification-free long-read sequencing of TCF4 expanded trinucleotide repeats in Fuchs Endothelial Corneal Dystrophy

**DOI:** 10.1371/journal.pone.0219446

**Published:** 2019-07-05

**Authors:** Eric D. Wieben, Ross A. Aleff, Shubham Basu, Vivekananda Sarangi, Brett Bowman, Ian J. McLaughlin, John R. Mills, Malinda L. Butz, Edward W. Highsmith, Cristiane M. Ida, Jenny M. Ekholm, Keith H. Baratz, Michael P. Fautsch

**Affiliations:** 1 Department of Biochemistry and Molecular Biology, Mayo Clinic, Rochester, Minnesota, United States of America; 2 Division of Biostatistics and Bioinformatics and Department of Health Sciences Research, Mayo Clinic, Rochester, Minnesota, United States of America; 3 Pacific Biosciences of California, Inc., Menlo Park, CA, United States of America; 4 Department of Laboratory Medicine and Pathology, Mayo Clinic, Rochester, MN, United States of America; 5 Department of Ophthalmology, Mayo Clinic, Rochester, Minnesota, United States of America; University of Florida, UNITED STATES

## Abstract

Amplification of a CAG trinucleotide motif (CTG18.1) within the TCF4 gene has been strongly associated with Fuchs Endothelial Corneal Dystrophy (FECD). Nevertheless, a small minority of clinically unaffected elderly patients who have expanded CTG18.1 sequences have been identified. To test the hypothesis that the CAG expansions in these patients are protected from FECD because they have interruptions within the CAG repeats, we utilized a combination of an amplification-free, long-read sequencing method and a new target-enrichment sequence analysis tool developed by Pacific Biosciences to interrogate the sequence structure of expanded repeats. The sequencing was successful in identifying a previously described interruption within an unexpanded allele and provided sequence data on expanded alleles greater than 2000 bases in length. The data revealed considerable heterogeneity in the size distribution of expanded repeats within each patient. Detailed analysis of the long sequence reads did not reveal any instances of interruptions to the expanded CAG repeats, but did reveal novel variants within the AGG repeats that flank the CAG repeats in two of the five samples from clinically unaffected patients with expansions. This first examination of the sequence structure of CAG repeats in CTG18.1 suggests that factors other than interruptions to the repeat structure account for the absence of disease in some elderly patients with repeat expansions in the TCF4 gene.

## Introduction

Fuchs Endothelial Corneal Dystrophy (FECD) is a late-onset degenerative disease of the cornea that affects approximately 4% of the Caucasian population in the United States. More than 75% of the cases of FECD are strongly associated with the expansion of a CAG (CTG18.1) [1] repeat in the TCF4 gene. Thus, FECD is the most common disease that is attributable to the expansion of a trinucleotide repeat.

In our work with FECD, we have noted that a small minority of elderly individuals with expansions of the CTG18.1 repeat do not develop FECD [2]. In several other repeat expansion diseases, it has been found that interruptions to the regular repeated sequence can reduce the penetrance of the associated phenotype [3–5]. To determine if interruptions within expanded CAG repeats exist in individuals who do not have FECD, we sought to sequence through the length of the repeat region in these samples.

One limitation of most strategies developed for sequencing trinucleotide repeat regions is the requirement for PCR amplification. PCR amplification of GC-rich repeats has the potential to create artefactual variants in the sequencing data due to polymerase slippage within the repeat region. Accordingly, we have used a modified version of the recently-developed No-Amp Targeted Sequencing method [6–8] that utilizes CRISPR-Cas9 in conjunction with Single Molecule, Real-Time (SMRT) sequencing to interrogate the sequence structure of CTG18.1 repeats in samples obtained from FECD and control patients. This method permits the enrichment and direct sequencing of targeted sequences without PCR amplification. The long-read sequencing technology of the PacBio (Pacific Biosciences of California, Inc., Menlo Park, CA) platform also permits the generation and analysis of full-length sequences from even expanded repeats.

Our sequence data do not reveal any novel interruptions in the CAG repeat structure of CTG18.1 expanded repeats in clinically unaffected individuals. However, they do reveal that some samples contain novel variation in the AGG repeats that immediately precede the CAG repeats. The implications of these findings for the pathogenesis of FECD are discussed.

## Methods

### DNA isolation and short-tandem repeat (STR) analysis

This study was approved by the Mayo Clinic Institutional Review Board (IRB) #06–007210. Research participants with and without FECD provided blood samples after written informed consent. The recruitment of subjects without FECD was limited to elderly individuals. Leukocyte-derived DNA was extracted using AutoGen FlexiGene by Qiagen (Valencia, CA). Repeats were PCR amplified as described previously [2] by incubating 100 ng of genomic DNA with 10 pmoles of oligonucleotide primers specific for TCF4 (5-TCF-Fuchs: 5’- CAGATGAGTTTGGTGTAAGATG-3’; 3-TCF-Fuchs 1: 5’-ACAAGCAGAAAGGGGGCTGCAA-3’) with Invitrogen Platinum PCR Super Mix High Fidelity (Carlsbad, CA). The PCR program was as follows: 100 ng of genomic DNA and 10 pmoles of each primer in 50 μl; Hot Start 95°C, 6 min. 1 cycle; then 95°C 1 min., 62°C 1 min., 68°C 3 min. for 35 cycles; 68°C 7 min. 1 cycle; and a 4°C hold.

For STR analysis, a 5’ FAM primer (5-FAM-TCF-Fuchs– 5’-CAGATGAGTTTGGTGTAAGATG-3’) was used instead of 5-TCF-Fuchs and PCR was performed as described above. After PCR amplification, GeneScan analysis was performed by GeneWiz (GeneWiz Corporation, South Plainfield, NJ).

### Testing guide RNAs (gRNA)

The TCF4 region was amplified by PCR using extended PCR using Takara LA Taq Kit 2.1 (Cat# RR013A; Tokyo, Japan) with Takara LA Taq Hot Start (Cat # RR042A) and the GC1 buffer supplied with the kit. Primer locations are shown in S1 Fig. The following PCR program was used: 95°C 2 min. 1 cycle; 95°C 1 min., 66°C 1 min., 72°C 10 min. for 35 cycles; 72°C 10 min. 1 cycle; and a 4°C hold. Following electrophoresis, the DNA product was extracted from agarose gel using Promega Wizard SV Gel and PCR Clean Up System (Cat. # A9281; Madison, WI). The product was cloned using Thermo Fisher Invitrogen Topo TA Cloning Kit Dual Promoter, with One Shot Top 10 chemically competent E. coli cells. (Cat. # K460001; Waltham, MA). Design of CRISPR RNAs (crRNAs) for experiments was performed using BROAD Institute’s Genetic Perturbation Platform gRNA design tool (https://portals.broadinstitute.org/gpp/public/analysis-tools/sgrna-design). crRNAs were selected based on the predicted on and off target efficacy scores as well as positioning relative to restriction enzyme sites. The crRNA were complexed with tracrRNA to form TCF4-specific gRNAs. The gRNAs were tested using cloned TCF4 DNA and the gRNA that most efficiently cut the cloned DNA was used for further genomic CRISPR/Cas9 experiments. Oligonucleotides comprising the gRNA (crRNA and tracrRNA) were obtained from Integrated DNA Technologies (Skokie, IL) containing an Alt-R modification to prevent RNase degradation. Cas9 nuclease *(S*. *pyogenes)* was obtained from New England Biolabs (Ipswich, MA).

DNA digested with crRNA in CRISPR/Cas9 protocol was analyzed using Advanced Analytical (AATI) fragment analyzer (Agilent; Santa Clara, CA). The High Sensitivity Large Fragment 50 Kb Kit (Agilent, catalog # DNF-464-0500) was used to analyze cloned digested products.

### Sanger sequencing

Amplified PCR products were sequenced by GeneWiz. PCR products were purified using Wizard SV Gel and PCR Clean up System (Promega, Madison, WI). DNA concentration was determined using Qubit. DNA and primer were combined as follows: 40 ng of DNA and 25 picomoles of primer in a final volume of 15 μl.

### No-Amp Targeted Sequencing

Purified genomic DNA (20 μg) was digested with MfeI restriction enzyme (New England Biolabs) to isolate the region of interest in a 6.7 kb fragment. A SMRTbell library was prepared from the MfeI fragments by ligation for 16 hours at 16°C with a hairpin adapter containing an overhang sequence complementary to the MfeI cut site using *E*. *coli* DNA ligase (New England Biolabs). Genome complexity reduction was performed by incubating each sample with a mixture of restriction enzymes BamHI-HF and XhoI HF plus Exonuclease III and Exonuclease VII (New England Biolabs). Up to 1 μg of the complexity-reduced SMRTbell library for each sample was subjected to Cas9 digestion with a single gRNA specific to the sequence adjacent to the target region. Oligonucleotides comprising the gRNA (crRNA and tracrRNA) and Cas9 nuclease (*S*. *pyogenes)* were obtained from Integrated DNA Technologies and New England Biolabs respectively (see above). Cas9-digested SMRTbell templates were ligated for 16 hours at 16°C with a poly(A) hairpin adapter using T4 DNA ligase (Thermo Fisher Scientific, Waltham, MA) producing asymmetric SMRTbell templates. Failed ligation products were removed by treatment with Exonuclease III and Exonuclease VII (New England Biolabs). Asymmetric SMRTbell templates were enriched with MagBeads and buffers from MagBead Kit v2 (Pacific Biosciences). In preparation for sequencing, a standard PacBio sequencing primer, lacking a poly(A) sequence, was annealed to the enriched SMRTbell templates in diluted Primer Buffer v2 (Pacific Biosciences). Sequel DNA Polymerase 2.1 was bound to the primer-annealed SMRTbell templates with reagents from the associated Sequel Binding Kit 2.1 (Pacific Biosciences). The sample complex was purified using AMPure PB reagent (Pacific Biosciences). Each purified sample complex was loaded on a separate single SMRT Cell (Pacific Biosciences) and sequenced on a Sequel System (Pacific Biosciences) using an immobilization time of 4 hours and movie time of 10 hours.

### Data analysis

The sequencing data were analyzed with RepeatAnalysis (RA), a new tool for analyzing SMRT sequencing data from target enrichment experiments (Pacific Biosciences). In brief, the tool performs a fast heuristic search using Sparse Dynamic Programming (SDP) alignment [9] to identify which reads belong to which target loci from a list supplied by the user in FASTA format. After grouping by targeted loci, the approximate repeat sizes were estimated by performing a classic Smith-Waterman [10] alignment against *in silico* mutated template sequences with varied tandem repeat counts. Using those approximate size estimates as the starting point, more precise estimates were computed using the slower, but more sensitive Quiver model [11]. The log-likelihood probabilities Quiver model was then aggregated by zero-mode wavelength and converted to relative probabilities, so all unique molecules were weighted equally. Finally, per-molecule probabilities were combined and the minimum set of unique, high-scoring templates that best explain the data to the user were selected.

The alignment-free workflow for extracting TCF4 repeat sequence from circular consensus sequence (CCS) reads and its visualization follows the protocol described in Hoijer and colleagues [7]. Briefly, the TCF4 gene contains CAG (AGC) repeats (chr18:53,253,385–53,253,459) with upstream AGG repeats (Chr18:53,253,349–53,253,384). Fifteen bp of flanking sequence upstream of AGG (chr18:53,253,334–53,253,348, sequence: “AAGAAGGTCTAGAAG” underlined in S1B Fig) and downstream of CAG (chr18:53,253,460–53,253,474, Sequence: “ATGAAAGAGCCCCAC” underlined in S1B Fig) were used as anchor sites for retrieving CCS reads that contain both sequences (allowing two base mismatches) thereby filtering out non-specific reads and increasing robustness of downstream analysis. The CCS reads were then trimmed to the point of containing only the repeat sequence. An additional step for indel correction was performed on repeat units that contained possible sequencing errors and flanked by correct repeat units, as described in Hoijer and colleagues [7]. Finally, each of the extracted and corrected repeat sequences were plotted horizontally in a histogram with trinucleotide repeats colored red for AGC and blue for AGG with interruptions and deviations colored in grey. All genomic coordinates are based on GRCh37 (hg19) genome sequence.

## Results

### Application of the No-Amp Targeted Sequencing method to CTG18.1

The restriction enzyme MfeI was used as the primary enzyme for the No-Amp Targeted Sequencing method. Digestion with Mfe1 produced a 6.7 kb DNA fragment that spans the CTG18.1 repeat region (S1 Fig) and was compatible with previously-designed EcoRI target adapters required for the No-Amp method [6].

**Fig 1:**
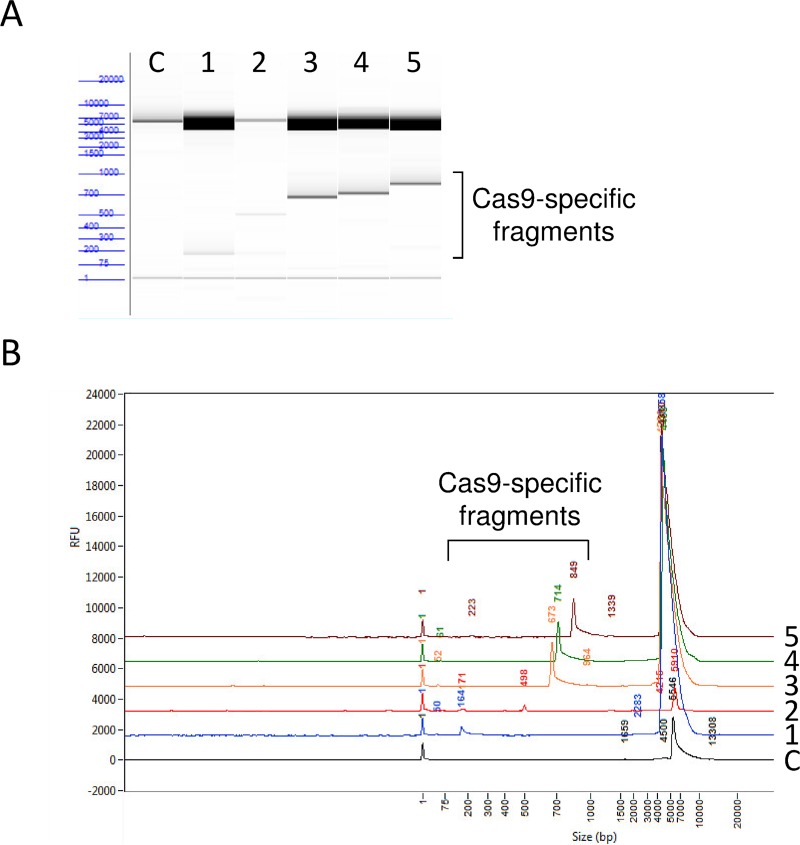
Fragment analysis testing of 5 different gRNA designs. **a.** Digital gel image of products resulting from Cas9 digestion of CTG18.1 plasmid with five different gRNAs (lanes 1–5). No gRNA was added to the sample shown in lane C. **b.** Trace view of same data shown in (**a**), giving specific sizing for the Cas9 digestion products with each gRNA.

To optimize the selection of gRNA sequences for the interrogation of the CTG18.1 repeats in TCF4, we cloned a 2.4 kb segment of the TCF4 gene surrounding the repeats into the Topo TA vector and used it as a substrate for testing candidate gRNAs. As shown in Fig 1A, 3 of the 5 candidate gRNAs (3–5) led to specific and efficient cutting by Cas9 as evidenced by the appearance of the predicted digestion products and verified by a fragment analyzer (Fig 1B). Based on these results, we selected gRNA cr3 for use with our genomic DNA samples.

**Fig 2:**
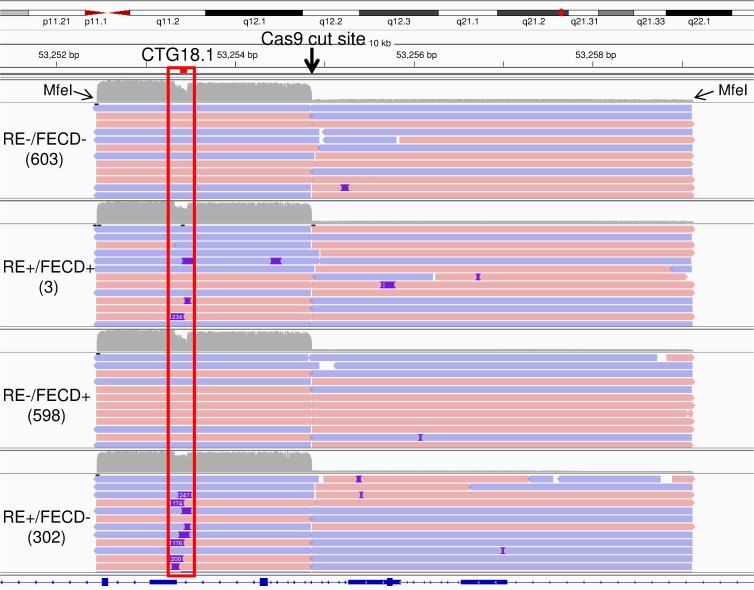
Integrated Genomics Viewer screenshot of a 10kb region surrounding CTG18.1 in the TCF4 gene. Representative samples from our four classes of FECD and control samples are shown. The location of the CAG repeats is shown by the red bar above the top sequence track. The locations of the flanking MfeI sites are noted, as is the Cas9 cut site. Insertions are shown as blue marks on individual sequence reads.

After targeted digestion of genomic DNA with Cas9, the 6.7 kb MfeI fragment was cleaved to a 2.4 kb fragment containing the CAG repeat region and 4.3 kb fragment containing upstream sequences. Fig 2 shows a screenshot from Integrated Genomics Viewer (IGV) of PacBio sequencing reads from a 10 kb region surrounding the targeted repeats. Results from 4 different samples demonstrate the robust recovery of the targeted region using this method. The four samples represent four categories of samples that we have characterized during our studies of CTG18.1 repeat expansions in FECD (Table 1). In addition to the clinical diagnosis of FECD (FECD+ and FECD-), we have separated samples into those that have CTG18.1 repeat lengths less than 40 which is the clinical threshold for expansion (RE-) and those that have expansions ≥ 40 (RE+). Using these designations, unaffected controls without repeat expansions were designated as RE-/FECD- (Fig 2, sample 603), and samples with repeat expansions and FECD are designated RE+/FECD+ (Fig 2, sample 003). The third category represents RE-/FECD+, the minority of patients whose FECD is attributed to variants in other genes (Fig 2, sample 598). The final category of RE+/FECD- have repeat expansions but have not developed clinical signs of FECD (Fig 2, sample 302). The average coverage for all 18 samples studied was 582X (range 40X-2095X), which was more than sufficient to evaluate the sequence structure of the CTG18.1 repeats in all samples (Table 1). We did not systematically investigate the variability of the coverage but believe it reflects the quality of the individual DNA samples and some variability due to target capture and the washing of the captured targets.

**Table 1:** Demographics, FECD and CTG18.1 repeat status of samples.

			Clinical grading (Krachmer Scale)		STR assay		Southern blot	PacBio		
Repeat and FECD Status	Sample ID	Age	OS	OD	short	long		short	long	Reads on target
RE+/FECD+	4938	74	6	6	26	89		27	50–1200*	840
RE+/FECD+	1	72	6	6	19	61		19	64	1103
RE+/FECD+	3	74	6	6	12	90		13	93	1327
RE+/FECD+	16	60	6	6	24	77		24	79	2095
RE+/FECD+	18	72	4	6	19	88		19	129	1647
RE+/FECD+	21	59	4	5	15	72		16	75	198
RE+/FECD+	23	64	6	6	16	74		17	75	1315
RE+/FECD+	4823	81	6	6	12	112	up to 8 kb	12,15	120–150*	131
RE+/FECD+	37	60	6	6	18	132	up to 8 kb	19	130–500*	170
RE+/FECD-	337	82	0	0	17	82		18	83	119
RE+/FECD-	554	81	0	0	12	67		13	68	262
RE+/FECD-	180	91	0	0	19	84		18	85	71
RE+/FECD-	302	68	0	0	18	92		19	122	460
RE+/FECD-	677	63	0	0	12	103		13	100–300*	88
RE-/FECD-	663	82	0	0	18	30		19	31	183
RE-/FECD-	603	79	0	0	12	15		13	16	182
RE-/FECD+	598	92	6	6	12	16		13	17	255
RE-/FECD+	26	85	6	6	12	16		13	17	40

RE+/FECD+; TCF4 trinucleotide repeat expansion and Fuchs endothelial corneal dystrophy

RE+/FECD-; TCF4 trinucleotide repeat expansion and no Fuchs endothelial corneal dystrophy

RE-/FECD-; no TCF4 trinucleotide repeat expansion and no Fuchs endothelial corneal dystrophy

RE-/FECD+; no TCF4 trinucleotide repeat expansion but has Fuchs endothelial corneal dystrophy

STR; short tandem repeat

OS; left eye

OD; right eye

*Zero-Mode Wavelength data

As can be seen in Fig 2, there is reduced coverage in the immediate vicinity of the repeats. This likely reflects the fact that the shorter allele in each of the samples is smaller than the 26 repeats found in the reference sequence, as measured by our standard PCR-based STR assay for repeat length (Table 1). Two samples in this figure have repeat expansions (3 and 302). These are detected as clear insertions in some of the PacBio reads (shown as blue rectangles within the individual reads).

For each of the 18 samples shown in Table 1, we determined CAG repeat length by our standard PCR-based STR assay. Southern blotting was used to investigate the possibility of very long expansions in samples that yielded only a single allele in the STR assay. The results of these assays have been used to assign each sample to one of the four groups shown in Table 1. Agreement between the STR assay and the No-Amp results was high for the smaller, non-pathogenic alleles, and for the smaller pathogenic alleles (less than 85 repeats) showing a difference of only a single repeat in most cases. For alleles with more than 85 repeats, there was more difference between the STR results and the No-Amp method. In some cases, the software used to analyze the PacBio data did not provide an automated call. Further analysis of the expanded repeats suggests that this discrepancy was due to substantial heterogeneity in repeat expansion length in these samples (see below).

### Sequence structure of the CTG18.1 repeats in FECD and control samples

**Fig 3:**
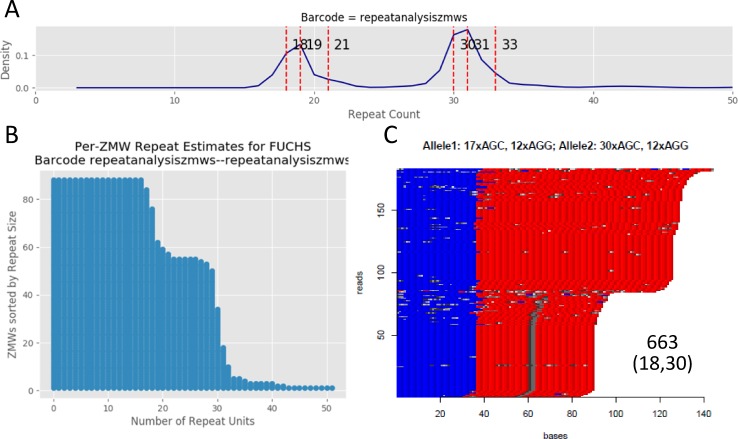
Analysis of trinucleotide repeats by long-read SMRT sequencing for RE-/FECD-. **a.** CAG repeat lengths in representative RE-/FECD- sample 663. Repeat count histogram showing two peaks at 19 and 31 repeats agrees well with the results of STR analysis (18 and 30 repeats). **b.** CAG repeat lengths versus the number of zero-mode waveguides (ZMW) that support the read length for sample 663. **c.** HTT repeat analysis plot of sample 663. Perfect AGG repeats are shown in blue, and perfect CAG repeats are shown in red. Other trinucleotide sequences in the target region are show in grey. Note the interruption of the CAG repeats in the smaller allele, shown as a vertical grey track.

The primary sequence data for the samples shown in Table 1 was analyzed by two separate software tools, the PacBio RA and the HTT-repeat analysis tool. Using the RA tool, we found high concordance between the No-Amp results and the STR results for both RE- samples and RE+ samples. For example, sample 663 is RE-/FECD- and has STR measured alleles of 18 and 30 repeats. With RA analysis, the measured peaks are at 19 and 31 repeats (Fig 3A). Similarly, the HTT-repeat analysis tool showed concordance with this assessment, highlighting two alleles at 17 and 30 repeats (Fig 3B and red region in Fig 3C). Importantly, the HTT tool also demonstrated the effect of a disruption in the regular CAG repeat structure. As was noted previously [12], a minority of our samples (including 4 of the 18 studied: 001, 018, 180 and 663; see Fig 1 and S2 Fig) do possess a G>C variant at chr18:53,253,431 (GRCh37, rs143743309, creating a CAC instead of a CAG). This is in agreement with our previous analysis that noted this particular interruption is not limited to either FECD+ or FECD- samples. The small allele in 663 shows this interruption, visible as a vertical gray bar in the bottom 60 reads (Fig 3C). We note that in this population of samples, the CAC variant is always seen on an allele with less than 20 repeats. The RA tool also revealed two reads that extend just into the pathogenic range (>40 repeats, Fig 3B). This is not shown in the HTT repeat analysis tool output, where the longest alleles are approximately 140 bp in length. Subtracting 36 bp for 12 AGG repeats (blue), this predicts about 35 repeats for the longest CAG-repeat products. Also notable in the HTT-repeat analysis tool output is the length and structure of the AGG repeats that immediately flank the CAG repeats (shown in blue in Fig 3C). In sample 663, there are 12 AGG repeats in each allele.

**Fig 4:**
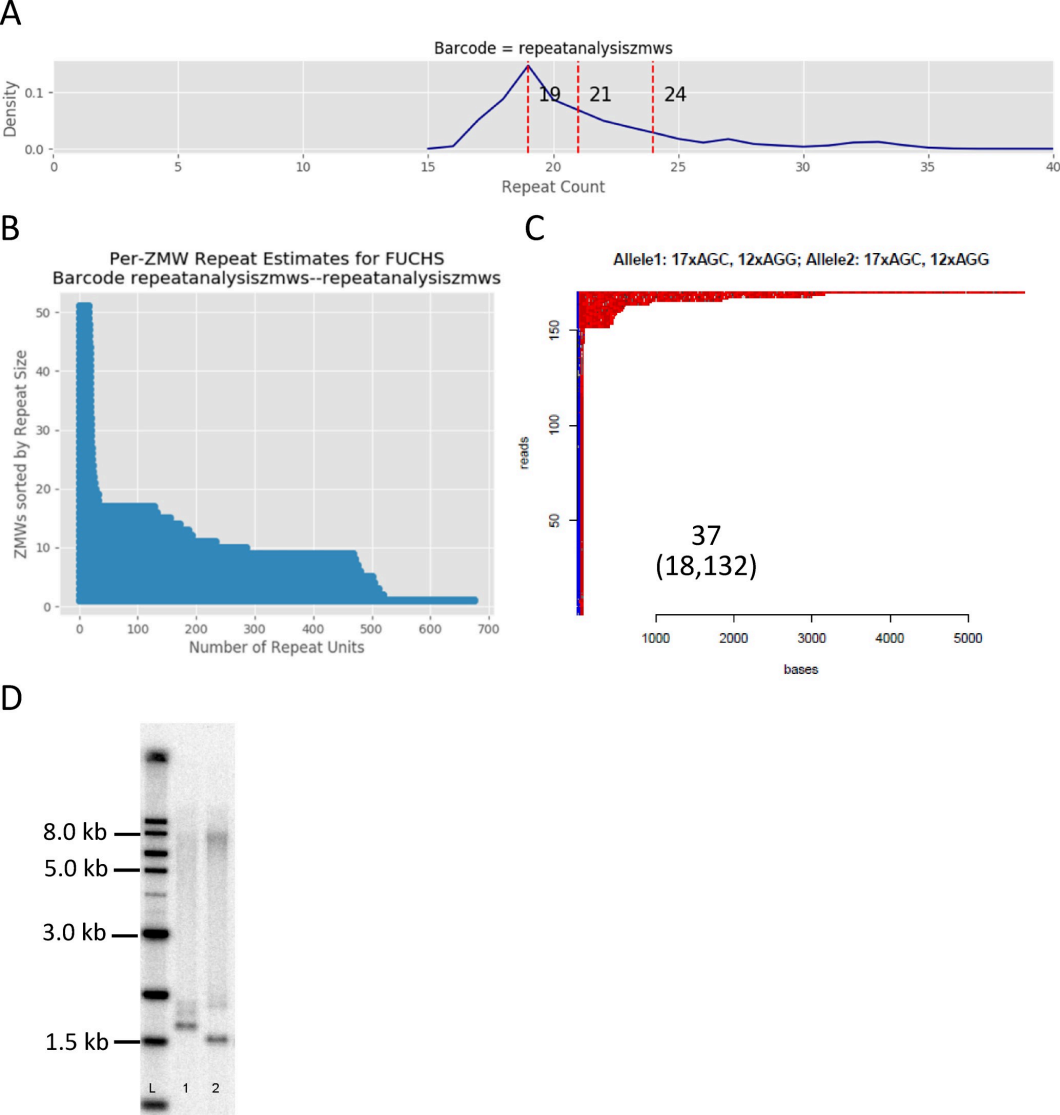
Analysis of trinucleotide repeats by long-read SMRT sequencing for RE+/FECD+. **a.** Repeat count histogram for representative RE+/FECD+ sample 37. Only the smaller allele (peak at 19 repeats) is detected by this software at this depth of coverage. **b.** CAG repeat length versus ZMWs for sample 37. **c.** HTT repeat analysis plot of sample 37. Blue and red areas depict AGG and CAG repeats as described in Fig 1C. **d.** Southern blot of 37 (lane 2), showing repeat expansion fragments up to 8 kb. Lane 1 is another RE+/FECD+ sample. Lane L contains radioactive size markers as indicated.

Looking across all samples (S2 Fig), the results reveal a remarkable heterogeneity in the size of expanded alleles. Given that these samples were not PCR amplified, this suggests somatic instability of the expanded repeat sequence and consequent mosaicism within the population of leukocytes used for the analysis of each specimen. In contrast, there is very little heterogeneity of subpathogenic alleles (<40 repeats). For example, in sample 37 (Fig 4A), the smaller allele is measured at 19 repeats by this analysis software and the distribution of alleles around this number of repeats is very tight (Fig 4B and 4C). However, there appear to be at least three categories of expanded repeats for this sample. The expanded repeats begin at approximately 120 repeats with another group identified at approximately 480 repeats. A single read with roughly 680 repeats was also identified at this level of sampling. These findings are consistent with the STR analysis, with a set of peaks at 132 repeats, and Southern blotting (Fig 4D) identifying a heterogeneous group of products extending to over 2000 repeats (roughly 8 kb including the flanking sequences defined by EcoRI digestion).

Using the HTT-repeat analysis software tool [7] for analysis, the lack of variability in the smaller allele for 37 is emphasized (Fig 4C). However, there is heterogeneity in the expanded alleles. There is a bolus of repeats in the range of 400–800 bp (~130–250 repeats) and an even longer set of reads extending from 2000 to over 5000 bp (up to 1600 repeats; Fig 4C), consistent with results obtained from Southern blots (Fig 4D). Similar profiles were observed in the other samples with repeat expansions greater than a few hundred repeats (S2 Fig).

**Fig 5:**
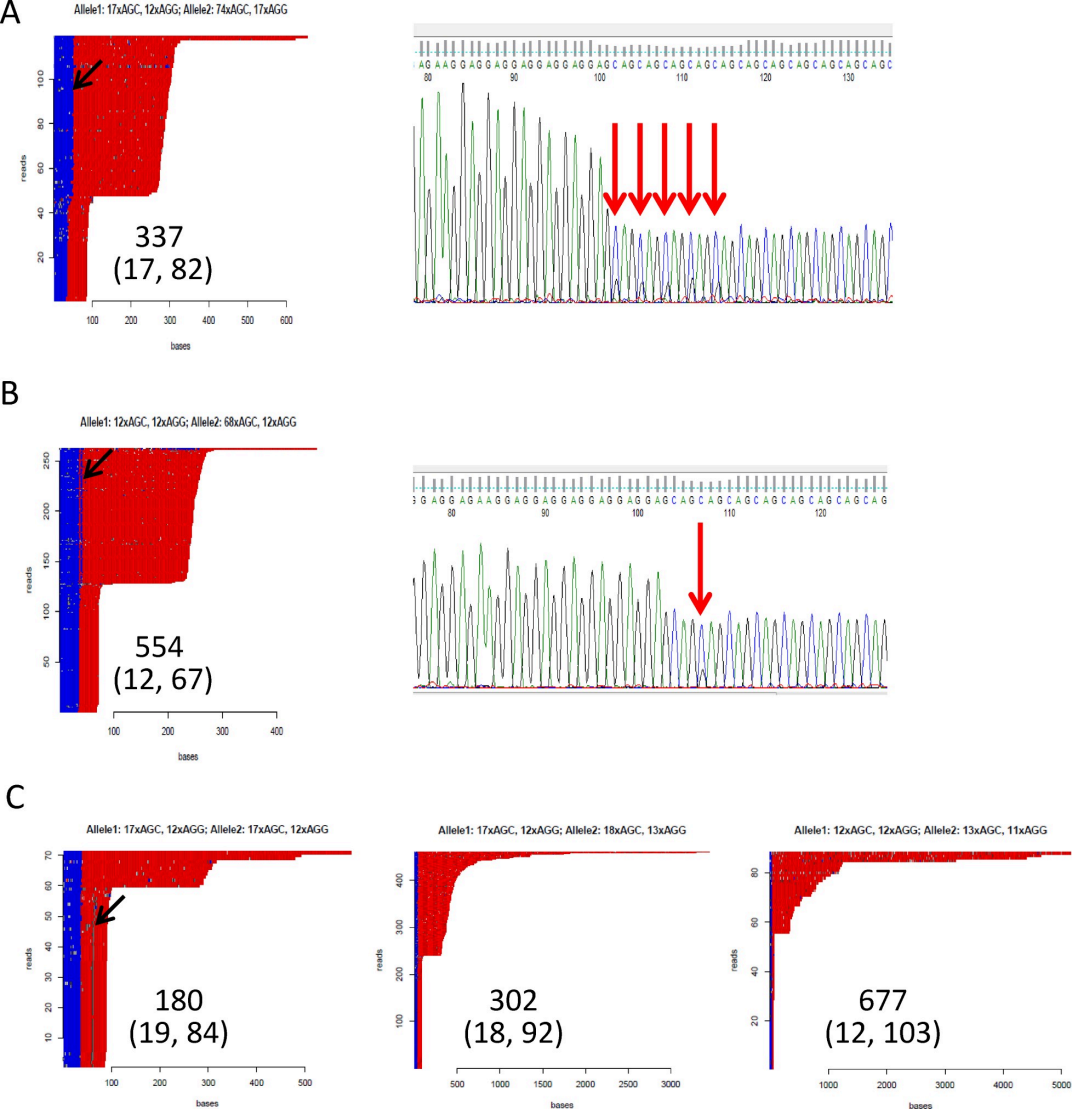
HTT repeat analysis plots for RE+/FECD-. **a.** Sample 337 contains an expansion of the AGG tract in the larger allele (black arrow) and Sanger sequencing confirmed sequence differences that extend the AGG repeat (red arrows). **b.** Sample 554 contains a CAG interruption of the AGG repeats (black arrow) that was confirmed by Sanger sequencing (red arrow). **c.** HTT repeat analysis for the remaining three RE+/FECD- samples. Sample 180 has an interruption in the CAG repeats in the smaller allele (black arrow), but no additional interruptions in either the AGG or CAG repeats were noted. Note the presence of CAG repeats greater than 4 kb in samples 302 and 677.

Importantly, none of the 5 RE+/FECD- samples studied with the No-Amp method possessed sequencing imperfections or interruptions in all of their expanded CAG repeats (Fig 5). Each clearly shows one shorter allele and one expanded allele (ranging from 67 to 103 repeats as measured by STR analysis) without a consistent interruption of a regular CAG repeat structure in the expanded alleles. However, two of the five RE+/FECD- samples (554 and 337) do have variant sequences within the AGG repeat that immediately flanks the CAG repeats in TCF4. The expanded allele of sample 337 (Fig 5A) has 17 AGGs instead of the 12 found in the reference sequence, and the expanded allele of sample 554 (Fig 5B) has the 12 AGGs found in the reference, followed immediately by a single CAG triplet and then one more (13^th^) AGG. Interestingly, both of these variant AGG sequences are found on the allele with the CAG repeat expansion, and both of these variants were verified by Sanger sequencing (see red arrows in Fig 5A and 5B). Neither of these two variants were identified in the other 3 RE+/FECD- samples (Fig 4C) although sample 180 does have an interruption in the CAG repeats of the smaller allele.

## Discussion

Amplification-free targeted enrichment in conjunction with long-read SMRT Sequencing (No-Amp Targeted Sequencing) was used to examine the sequence structure of CTG18.1 CAG repeat expansions in the TCF4 gene that are strongly associated with FECD. The ability to interrogate native DNA sequences in complex regions of the genome has provided several novel insights into the structure of the CTG18.1 repeats and surrounding sequences in RE+/FECD+ and RE+/FECD- samples. Hafford-Tear and colleagues [13] recently reported using No-Amp Targeted Sequencing to examine CTG18.1 repeat structure in a group limited to two RE-/FECD+ and nine RE+/FECD+ samples, but no previous detailed studies of CTG18.1 in RE+/FECD- or RE-/FECD- samples were reported.

From a technological perspective, No-Amp Targeted Sequencing yields data that highly agrees with results of the STR assay that we have commonly used to size the unexpanded or moderately-expanded CAG repeats at this locus. Similar to the STR assay, heterogeneity in the sizing of expanded repeats is also seen with the No-Amp method, with the most extreme sample (4938) exhibiting a range from 50 to 1200 repeats. This was also noted by Hafford-Tear and colleagues [13] and supports the hypothesis that heterogeneity of the repeat size is most likely biological in origin rather than a technical artifact of PCR. The possibility that the observed heterogeneity from the No-Amp method is due to polymerase stuttering on individual templates is countered by the lack of significant heterogeneity in the samples with sub-pathological repeat sizes (RE- samples in Table 1). All of the No-Amp size estimates for sub-pathological length repeats match the STR results within 1 repeat triplet.

Analysis of the No-Amp results with the HTT-repeat analysis software clearly identifies disruptions within the regular repeat structure, as evidenced by the confirmation of a CAC variant in the unexpanded allele of four of the samples studied here. The finding that this disruption was found only in unexpanded alleles is interesting and agrees with the hypothesis that this interruption in the CAG repeats might help to protect against expansion to the pathological range. In spinocerebellar ataxia type 1, the observation that 98% of unexpanded alleles have an interruption, but most expanded alleles have pure CAG repeats, has suggested that the loss of interruptions might contribute to expansion and subsequent pathogenesis of the phenotype [14]. Similar conclusions have been made in the case of CGG repeats in Fragile X syndrome [15].

Significantly, we did not find any evidence of reproducible sequence disruptions of the expanded CAG repeats in the five RE+/FECD- samples examined in this study. These findings argue against the hypothesis that interruptions to the CTG18.1 CAG repeat structure explain the absence of FECD phenotype in these elderly subjects that have CAG repeat expansions within the pathological range. However, we cannot exclude the possibility that interruptions to CAG repeats may occur in a subset of RE+/FECD- reads and those might contribute to reduced penetrance of the repeat expansion and no FECD. We also acknowledge that our sample size was small and some RE+/FECD- individuals may in fact have interrupted CAG repeats.

Our data also confirm that some RE+/FECD- samples have very large CAG expansions in DNA from leukocytes (up to thousands of repeats), implicating a mechanism that protects these individuals from developing FECD, independent of repeat expansion size. Of course, due to technical limitations, we have been unable to directly assay the CAG repeat length in corneal endothelium. Given the somatic instability of repeat length in leukocyte DNA, it is quite feasible that length and distribution of CAG repeats may be different in corneal endothelial tissue. However, we favor the hypothesis that other genetic or environmental factors contribute to the absence of disease in these RE+/FECD- patients rather than contraction of the repeat length in the cornea. Recent RNASeq studies investigating this question suggest that the RE+/FECD- samples lack some of the splicing disruptions that are considered to be characteristic in RE+/FECD+ samples (manuscript submitted).

The No-Amp results also revealed previously unreported variants within the AGG repeats that immediately follow the CAG repeats. The reference sequence contains 12 AGG repeats but others have noted heterogeneity in the length of the AGG repeats. Most recently, Hafford-Tear et al [13] reported sizes for these repeats between 8 and 15 repeat units. We found that two of the five RE+/FECD- samples had unique variants to the AGG repeat, including a 17 AGG repeat allele on the expanded CAG allele in sample 337 (Fig 5A). The heterogeneity of the AGG sequence has not routinely been accounted for in the STR sizing of the CAG repeats, but could presumably be significant in the sizing of borderline CAG repeat alleles. Thus, as suggested by Hafford-Tear et al. [13], sequencing through this region might be indicated for such samples in the future.

In summary, the PacBio No-Amp Targeted Sequencing method has provided new insights in the structure of the CAG and AGG repeats found in the CTG18.1 locus within the human TCF4 gene. No interruptions to the CAG repeat structure of expanded alleles in RE+/FECD- samples were identified, but two of five RE+/FECD- samples did have novel structures within the AGG repeats that immediately flank the CAG repeats. Heterogeneity in repeat length was confirmed by this No-Amp method, and the possibility of a broad range of repeat length within any given sample should be recognized in future efforts to size and interpret CAG expansions in relation to phenotypic expression.

## Supporting information

S1 Fig TCF4 sequence surrounding the CTG18.1 repeat. **a.** The MfeI sites used for the No-Amp Targeted Sequencing method are highlighted in green, and the sequences of the 5 candidate gRNAs are highlighted in aqua. The location of primers used to amplify the fragment used for cloning and testing the gRNAs are shown in red. The reference sequence in the vicinity of the CTG18.1 repeat is highlighted in yellow. **b.** The reference sequence context of the CTG18.1 repeats. Note that the reference sequence is the reverse complement of the sequence shown in 1a. The two flanking sequences used by the HTT-repeat analysis tool are underlined. The sequence between the AGG repeats and the 3’ flanking sequence is shown in bold. This sequence does contain the commonly-referenced CAG repeats, but is better described as a pure AGC repeat.
(PDF)

S2 Fig Assessment of repeat length by long-read SMRT sequencing for additional samples. The repeat count histograms, ZMW vs repeat length plots, and HTT repeat analysis plots are provided for each sample in this study as described in the legend to Fig 1. In each case, the STR results for that sample are given in parentheses immediately after the sample name.
(PDF)

## References

[1] Breschel TS, McInnis MG, Margolis RL, Sirugo G, Corneliussen B, Simpson SG, et al A novel, heritable, expanding CTG repeat in an intron of the SEF2-1 gene on chromosome 18q21.1. Hum Mol Genet. 1997;6(11):1855–63. 10.1093/hmg/6.11.1855 . 9302263

[2] Wieben ED, Aleff RA, Tosakulwong N, Butz ML, Highsmith WE, Edwards AO, et al A common trinucleotide repeat expansion within the transcription factor 4 (TCF4, E2-2) gene predicts Fuchs corneal dystrophy. PLoS One. 2012;7(11):e49083 Epub 2012/11/28. 10.1371/journal.pone.0049083 . 23185296 PMC3504061

[3] Matsuura T, Fang P, Pearson CE, Jayakar P, Ashizawa T, Roa BB, et al Interruptions in the expanded ATTCT repeat of spinocerebellar ataxia type 10: repeat purity as a disease modifier? Am J Hum Genet. 2006;78(1):125–9. 10.1086/498654 . 16385455 PMC1380209

[4] Stolle CA, Frackelton EC, McCallum J, Farmer JM, Tsou A, Wilson RB, et al Novel, complex interruptions of the GAA repeat in small, expanded alleles of two affected siblings with late-onset Friedreich ataxia. Mov Disord. 2008;23(9):1303–6. 10.1002/mds.22012 . 18464277

[5] Kraus-Perrotta C, Lagalwar S. Expansion, mosaicism and interruption: mechanisms of the CAG repeat mutation in spinocerebellar ataxia type 1. Cerebellum Ataxias. 2016;3:20 10.1186/s40673-016-0058-y . 27895927 PMC5118900

[6] Tsai Y-C, Greenberg D, Powell J, Hoijer I, Ameur A, Strahl M, et al Amplification-free, CRISPR-Cas9 Targeted Enrichment and SMRT Sequencing of Repeat-Expansion Disease Causative Genomic Regions. bioRxiv. 2017:203919. 10.1101/203919

[7] Hoijer I, Tsai YC, Clark TA, Kotturi P, Dahl N, Stattin EL, et al Detailed analysis of HTT repeat elements in human blood using targeted amplification-free long-read sequencing. Hum Mutat. 2018;39(9):1262–72. 10.1002/humu.23580 . 29932473 PMC6175010

[8] Ebbert MTW, Farrugia SL, Sens JP, Jansen-West K, Gendron TF, Prudencio M, et al Long-read sequencing across the C9orf72 'GGGGCC' repeat expansion: implications for clinical use and genetic discovery efforts in human disease. Mol Neurodegener. 2018;13(1):46 10.1186/s13024-018-0274-4 . 30126445 PMC6102925

[9] Baker BS, Giancarlo R. Sparse dynamic programming for longest common subsequence from fragments. J Algorithm. 2002;42(2):231–54. 10.1006/jagm.2002.1214 WOS:000175127900002.

[10] Waterman MS, Smith TF, Beyer WA. Some Biological Sequence Metrics. Adv Math. 1976;20(3):367–87. 10.1016/0001-8708(76)90202-4 WOS:A1976BY66800003.

[11] Chin CS, Alexander DH, Marks P, Klammer AA, Drake J, Heiner C, et al Nonhybrid, finished microbial genome assemblies from long-read SMRT sequencing data. Nature Methods. 2013;10(6):563–+. 10.1038/nmeth.2474 WOS:000319668700028. 23644548

[12] Wieben ED, Aleff RA, Eckloff BW, Atkinson EJ, Baheti S, Middha S, et al Comprehensive assessment of genetic variants within TCF4 in Fuchs' endothelial corneal dystrophy. Invest Ophthalmol Vis Sci. 2014;55(9):6101–7. 10.1167/iovs.14-14958 . 25168903 PMC4179444

[13] Hafford-Tear NJ, Tsai YC, Sadan AN, Sanchez-Pintado B, Zarouchlioti C, Maher GJ, et al CRISPR/Cas9-targeted enrichment and long-read sequencing of the Fuchs endothelial corneal dystrophy-associated TCF4 triplet repeat. Genet Med. 2019 10.1038/s41436-019-0453-x . 30733599 PMC6752322

[14] Chung MY, Ranum LP, Duvick LA, Servadio A, Zoghbi HY, Orr HT. Evidence for a mechanism predisposing to intergenerational CAG repeat instability in spinocerebellar ataxia type I. Nat Genet. 1993;5(3):254–8. 10.1038/ng1193-254 . 8275090

[15] Snow K, Tester DJ, Kruckeberg KE, Schaid DJ, Thibodeau SN. Sequence analysis of the fragile X trinucleotide repeat: implications for the origin of the fragile X mutation. Hum Mol Genet. 1994;3(9):1543–51. 10.1093/hmg/3.9.1543 . 7833909

