# Supplementary material for: Amplification-free long-read sequencing of TCF4 expanded trinucleotide repeats in Fuchs Endothelial Corneal Dystrophy
Source: PLoS One. 2019 Jul 5;14(7):e0219446. doi: 10.1371/journal.pone.0219446 (PMC6611681; doi:10.1371/journal.pone.0219446)
Supplement: S1 Fig — a. The MfeI sites used for the No-Amp Targeted Sequencing method are highlighted in green, and the sequences of the 5 candidate gRNAs are highlighted in aqua. The location of primers used to amplify the fragment used for cloning and testing the gRNAs are shown in red. The reference sequence in the vicinity of the CTG18.1 repeat is highlighted in yellow. b. The reference sequence context of the CTG18.1 repeats. Note that the reference sequence is the reverse complement of the sequence shown in 1a. The two flanking sequences used by the HTT-repeat analysis tool are underlined. The sequence between the AGG repeats and the 3’ flanking sequence is shown in bold. This sequence does contain the commonly-referenced CAG repeats, but is better described as a pure AGC repeat. (PDF) [file pone.0219446.s001.pdf]

Supplementary Figure 1

A

- **crTCF4-1 =** sgRNA: TGTCTGCGGATCTGTAGTGG (w/2.9 kb MfeI)
- **crTCF4-2 =** sgRNA: GGGACCGAGGACTTTGCCAG (w/2.6 kb MfeI)
- **crTCF4-3 =** sgRNA: AGCTACTTTGAAAAGCGGAG (w/2.4 kb MfeI)
- **crTCF4-4 =** sgRNA: GCGAGTGTGAGTGTGCGAGG (w/2.3 kb MfeI)
- **crTCF4-5 =** sgRNA: TAAAATGATGCGCTGAAGAG (w/2.2 kb MfeI)

CAATTGGATACCTTTAGATCCCAAACTTCATCCTATCATTGGTTTCTGGATAGATTATTTATTGCCCTTG
AAATTCAGCAGAAGGGGGCTGGGTGCAGTGGCTCACACCTGTAATTCCAGTACTTTGGGAGGCCGAGGTG
GGCAGACCACCTGAGGTCAGGGGTTTGAGACCACCCTGATGAACATGGAGAAATCCCATCTCTACTAAAA
ATACAAAATTAGCCGAGTGGGGTGACGCATGCCTGTAATCCCAGCTACTTGGGAGGCTGAGGCAGGAGAA
TCGCTTGAACCTGGGAGGTGGAGGTTGCAGTGAGCCAAGATCGTGCCATTGCACTCCAGCCTGGGCAACA
AGAGTGAAACTCCATCTCAAAACAAAACAAAACAAACAAACAAAAAAAGAAATTCAGCGGAATGAGGAGT
CTTAAAAACCTAAAAGCTAGAAGGGGTTTTATTGGATTATCTAATCTAACCTCTGGATTTTACAGCAAAA
GACATTGACATTGGGCCTTAGTTCAAAGAATGACTAGAGGTAGAGCCAGTGGAATTTCTCCTAACCCAAC
GTTTGTGATCTTTTCATTACATCATCTAAGCAGAACCTGTAGGGGTTAACTCTGAGAATTGGGGATGATG
CTTGTGATAATGCAGCAAAAAGTAATGTCTAAATGTAAGATATTATTTTGAGATGAGAACACCGAATGGG
GATGGAAGACAGGAATCTTCACCATGTGTGAATGTACAGGAAACAACCAATAAATGCCTTAATTCTTGTT
CTGTAAAAACTTACATGTAAGTAAAAGATCGCCGAAATTGATCTGAACTCTGTTACTGCCACAAACTTTT
ATCAGCAAATGGAGATTAGGATGTTCCCACTGCCAGATGGTATATAGGAATATGGTTGAATGTGGACTGT
TATCACAGACTGTCTGGTTTCAAAGGTGGGCTCTGTCACTCAATATAGTTGTGTGACTTAGTTAAGTTTG
TACCACTTACAAAACTTCTTCCTTGGGTCAAGCTACTCAATTTTACTTGCTTCTGTTGCCCAATATACAA
ATTACAGTAGTAATACATACTCCATAGGGTTATTCTTAGGCTTAAAGTAGCCAGTATTTGTGAAGTTATA
GGAACACTGTTTAGCATATAGTAAACACATGTGTTATCTGTCATTATTATTACTTGCTTTTCTGTTGCAA
AGACTAAAAGCTATTGTTTTGGAGTAGGGAAATTTCCATTTAAAATTCCATTGGCTAGGTTAGCGGTATC
AGTTGATTTAGCAAGGAATTAAGAGATGAAAAGGAATTATTTCTTTCGTGGTGGCTTCATCTTTATCTGT
ATCCACTATAAAATTCGTAAAGTGGGCGTTATTTGCTTTACACAGACCAGAAAACAGGCTACAGAAGGTA
ACTGCTTGGCCAGAAATGGGTTGAGTCAGTCGTGGCTTATGGCACAAACTACATTTGAGTCTACAAAGCA
ATAGATGAAGGTCTTCGCCAAGTGAAGACTTTTTGGACTTGGAAAGTTCACACACTTCCAGCGCTCTATA
AGAAGATAGGGTGGGGGAGTTGGAGGAGAGGACCCACATCCCTCTTCCAGCCTAGGACCAACTTGGCCCA
GAGGCCTTTTCCCATGCCGCCCAAGGCTCGCTGAGAAATCTGGCTCCACACAGTGGGAGAACCCAGGGTC
AGGCTTACTGTGAGTGAGGCCGGCCCAGTCTTTCCCGAGCCTTCCTCAGGGAGACCTGGGGGTATCTCAC
TTCCCTGCGGAAGTCTGTGGAGTTTAATTTGTAAATTTGTGCCGAAACTACACTTTTGTGTCAAAGAGCA
AGGACGGCCAAGAAGCGGCAACACGAATAAGAGCGCAGGGAAATGGGGCCTGCGAACGACCTCGGCCTCG
GGAACTCCCACAGTGCCGCCCCACGCGCGACGCCTACCCGAGAACAGCCACCCGCCTCCGCTCCCGGAGG
AGGCGTGGTCGACCAGCACCGCCATCTTGGCCGCCTCTCTAGGCGCTCTGTTTACCACTCTATGGTCGCC
TGCTCCCGCCCCGCCCCTTCCCAGTCATTGTCTGGAGGAGCAGCCGCGGCCGCAGCTCCTTCTCTTTATA
AGCCCGCAGTTCCCGGATGTGAATGGATTACAATGTATCTTTCAGGGAAACCTATTATTATCAATGTGAC
TCCACGGGGGAGTCCATGGTGATGATGATGAGGAGGAGGATGATGATGATGAGACACCTCTAAACTTGGA
ACAAGTTTAAGACTTTATGAGAGAAGAAAAAAAATCACCAACAAGAATTGTTTGAGGAAAAATTATAACT
ATCCTGTGTTCATTTTTTTTTTTATAAACAATAAGAAAAAGTTGTTGGATTTTTTTTTAATGATTTCTTT
TTTGGGGGAGGGAATTTTGTTGCAGTTTTATGGTGGAAAATGCAAAAACCAGAGCCAGGTGCATAATCTT
GTAATCTGTGGATATCCCTGGAGCAGGACTGAGTACCAGTTAAAATACTTTTTGGGGATACACATGTGAG
ATACTAAGTACTTGCAGAAGATTTTTGTCTCTCTTTTTAAAGTCTCTTTCCTTGGAATATTGTGAGAATA
TTTGTGGCCATTTAAGGTAACGTTTCAATTTGCCGTCAGAGTAAACTGTTTGTAATTGAATTTAATTTTT
AAAATGTCGATCCCGATGTTTTATTAAAACAAAAGGGACAACCTATTAAATAATTGCCAATGGGAAAAAG

TGTGTGCATGCATTTTGTGTATGTACTCGCACATCTAGCAATACCAGGATTTGTAGAATGTGTGTTTTTT
TTTTCAAACACACGTTAAAATAGCTTATGTTGCATATACCGGATAAAGCACGCTTGTTATTACGAAACGA
TAGAATTATGAAGCTTAAATTTTATCCTAAAAAGTGATGTGAAAAAGTATGTTTTAAGAGCATGCAGGGC
CTATAATGGTAAATGTTGAATTAAAAACTTACAGTGCTGGCGAACAGGGACTTAGCGTGCCTAGGAACTC
AAGTGCAAAAACAAAACAACATCCACCCCCAAAAATTGCCCTTTTTTTTTTTCGAAGTTTGCAGTTATAC
TATTCTGTGACTTAAAAGGGAGGGGAAAGAAAGTATGCAATTCAGATTTCGCCCCCAACGCAAAACTACC
GAAACGAAAATCTCCCCAGGAAAATGTGGCTTGATTTATAGACGAAAAAGAAAAATATTGTTCCTCTTGG
CAAAGTCTTTTTGCATCACGTGTATTTGCAGCCTAGTATAAACTTGCTTTGCCGTGTGTGGATGTGTGAG
TGAGAGGGAACGAGAGTAAGAGAAAGAAAGAAGTGAGGGGATGTAAACTCGAATAAATTTCAAAGTGCCT
CCGAGGGATGCAACGGGCAAAAACTGAACTGTTCAGGCTTCAGATTGTAACTGACGATCTGAGGAAAAAT
GAGGTGCTCGATGAATTTTCGTTTGTATTTTTTGGCGAGGCGGGGGAGGTGTTGAGATTTTTTTTTTTTC
CCCTCGGGGTGGGTGCGAGGGGGATGCATCCTAGCCTGCCCGACCCGGAGCAAGTCGCGTCTCCCCGCCG
GAGCCCC**CCCACCCATTTCTTTGCTGAAC**TTGCAATTCCGTGCGCCTCGGCGTGTTTCCCCCTCCCCCCT
TCCCTCCGTCCCCTCCCCTCCCCGGAGAAGAGAGTTGGTGTTAAGAGTCAGGGATCTTGGCTGTGTGTCT
GCGGATCTGTAGTGGCGGCGGCGGCGGCGGCGGGGAGGCAGCAGGCGCGGGAGCGGGCGCAGGAGCA
GGCGGCGGCGGTGGCGGCGGCGGTTAGACATGAACGCCGCCTCGGCGCCGGCGGTGCACGGAGAGCCCCT
TCTCGCGCGCGGGCGGTAGGTACCGGCGCCTGCGGGGCTCGGCGGGGCGGAGGCGCCCGGCGGCGCGGGG
TTCGGGCTCGGCGGCCCCGCACGCGGCTCCGCGCCTCCCGCGCCGCGGGCTCCCGGCGCCCGGCGCTCCC
AGAAGAGACACCCCTTCCCCTCCCGCCGCTTCCCTCCCCCTCGCCGCCAGCCCCCCCGCCCCTCCCCTTG
ATGCCCCCTCGGAGGGACCGAGGACTTTGCCAGGGGCCTGACTTTAATTTTTATAACCCCTTTCTTTCAC
AAATTAGGGTGCTGGACAATTAGAGGACCCGACCCTCCACTCCGCTCCCCCCAACCCTGTCACCCCACCC
CTCCCCCTTTGGACGTGTTTTCACTCCAGAGACGCTCATCTTTAATTTCTTGATAGCTACTTTGAAAAGC
GGAGGGGAGTGGTGTGTGCGAGTGTGTGTGCGTGTGCGAGTGTGAGTGTGCGAGGGGGGTGGGGTGCAGG
GTGGGGGGAAAGTTGAGATCGGATCGTTCTCAGTAGTTTCTCTTTTCTCTGTGATCCATTCATTTCTCGC
TCTACCTCCTGTTGCATAAAATGATGCGCTGAAGAGCGGCTTTTTTTTTTTCTTTCCCTGATTTGGTTTT
ATAGTGGATGTTACCAGTTTGATCGTCTCTTTGGAAATACAATATCGCTTTTTTTTTTTTTTTTTTTTT
TTTTTTTTTTTTTTTGCTATTGCTGCCTATTCCATCTTAAAAAAACAAAACCAAACCCAACCCAACAACA
AGTCTCCCATCCCTTCCTCCTTTCCACCTTAATCCCACCCGCCCCCACGTCCCCACCCCCAACCCCCCAG
TCTCCAAAAATCCGATTGTGTTTTCTCCAAGGATTGGGACTGGATTTTCTGATTGCGGTTATTTCCATGT
TTCTAGGTTTGTGTGATTTTGCTAAAATGCATCACCAACAGCGAATGGCTGCCTTAGGGACGGACAAAGA
GCTGAGTGATTTACTGGATTTCAGTGCGGTAAGAAAGAACGGTGGAAACTAACAACAGCTGTGAAAAAAA
CAAAACAAAAACCCAAACACTTCAGCTAGAAACCAGTAGGAATCTAAAGGACAGTAATAATTTTTAATTG
GCTGAATCCTTGGTAAATATGAAGGTCTTTTTGACAAGTTTTTAACTATAATTTTGTGGTGTGATGGAAG
ATTCAGGCTTTTTTTTTTTTTTTGAGTTTTATTACTGGCCTTCAATTCCCTACCCACTGATTACCCCAAAT
AATGGAATCTCACCCCAGTGGAAAGCAAAAATAGACACCCCTAAAACTAAACCACCCCTAAAACTTGGCC
ATGTCTGAACACTGAGACTACTAATACTTTGCACACTACTCTTCGTTTTATTTATTGTTTTTGGAAATGG
AAAATAGAAAATAGGAGACCCAGTTGTCTCTTTAAAGTTTTAAGCTAATGATGCTTTGGATTGGTAGGAC
CTGTTCCTTACATCTTACCTCCTAGTTACATCTTTTCCTAGGATTCTTAAAACTAGTATGGATATGCTGA
GCATACATTCTTTAGAACCTTTTGGACTGTTTTGGTAAATTTCGTAGTCGTAGGATCAGCACAAAGCGGA
ACTTGACACACTTGTGGAGTTTTACGGCTGTACTTGGTCCTTCTCCATCCCTTTGCTTCCTTTTCCTAAA
CCAAGTCCCAGACATGTCAGGAGAATGAATTCATTTTTAATGCCAGATGAGTTTGGTGTAAGATGCATTT
GTAAAGCAAAATAAAAAGAATCCACAAAACACACAAATAAAATCCAAACCGCCTTCCAAGTGGGGCTCTT
TCATGCTGCTGCTGCTGCTGCTGCTGCTGCTGCTGCTGCTGCTGCTGCTGCTGCTGCTGCTGCTGCTGCT
GCTGCTGCTCCTCCTCCTCCTCCTCCTTCTCCTCCTCCTCCTCCTCTTCTAGACCTTCTTTTGGAGAAAT
GGCTTTCGGAAGTTTTGCCAGGAAACGTAGCCCTAGGCAGGCAGCTTTGCAGCCCCCTTTCTGCTTGTTG
CACTTTCTCCATTCGTTCCTTTGCTTTTTGCAGGCTCTGA**CTCAGGGAAGGTGTGCATTATC**CACTAGAT
ACGTCGAAGAAGAGGGAAACCAATTAGGGTCGAAATAAATGCTGGAGAGAGAGGGAGTGAAAGAGAGAGT
GAGAGTGAGAGAGAGAGAGAGTCTTGCTTCAAATTGCTCTCCTGTTAGAGACGAAATGAGAATTTAGTGC
AGGTGGCACTTTTATTTTTATTTGGGTTCACATATGACAGGCAAATCCTATACGAGATGGAAATGGACAT
TGCCACGTTTATGGCCAAGGTTTTCAATATAAAACAAAACAACTTTTTTCTTCTCCTTGGTGAAACTAGT
GTTTTTCTAGAGAGGCTGCTGGCCTCCAACCTGAATCTTGATAACATTATGGGGACTGTGTTTGTTCCAA
ATGTAGCAGTAGTACTGCTTGGCCATCTAATGAACCTGAGGAAAAAGAAAGAACAGAGTGATAATGGGGG

CTGGGGTGGGATCTGTAATGTTGTTTCTCTTTTAGTTTTAAGTTGGATGGTGATGTATTTTACTAAATAA
ACCCTTAGCATAAACTCTAAGCTGTTTGGTAACAGTATGAAAGATCTTTGAGGAGCTCTGAAGGCACAAG
TGTCTTCTTTTCAACTGTAATATTTCTTTGTTTCTTTTAGATGTTTTCACCTCCTGTGAGCAGTGGGAAA
AATGGACCAACTTCTTTGGCAAGTGGACATTTTACTGGCTCAAGTATGTAAATTCTTTTCTTAGTGTTAA
GTTAAATGTTCTGGGCTGTTTATTTACTCAATTG

B

CCAAAAGAAGGTCTAGAAGAGGAGGAGGAGGAGGAGAAGGAGGAGGAGGAGGAGG**AGCAGCAGCAGCAGC**
AGCAGCAGCAGCAGCAGCAGCAGCAGCAGCAGCAGCAGCAGCAGCAGCAGCAGCAGCAGCATGAAAGAGC
CCCACTTGGAA
