# Supplementary material for: Amplification-free long-read sequencing of TCF4 expanded trinucleotide repeats in Fuchs Endothelial Corneal Dystrophy
Source: PLoS One. 2019 Jul 5;14(7):e0219446. doi: 10.1371/journal.pone.0219446 (PMC6611681; doi:10.1371/journal.pone.0219446)
Supplement: S2 Fig — The repeat count histograms, ZMW vs repeat length plots, and HTT repeat analysis plots are provided for each sample in this study as described in the legend to Fig 1. In each case, the STR results for that sample are given in parentheses immediately after the sample name. (PDF) [file pone.0219446.s002.pdf]

Supplementary Figure 2

001 (19, 61)

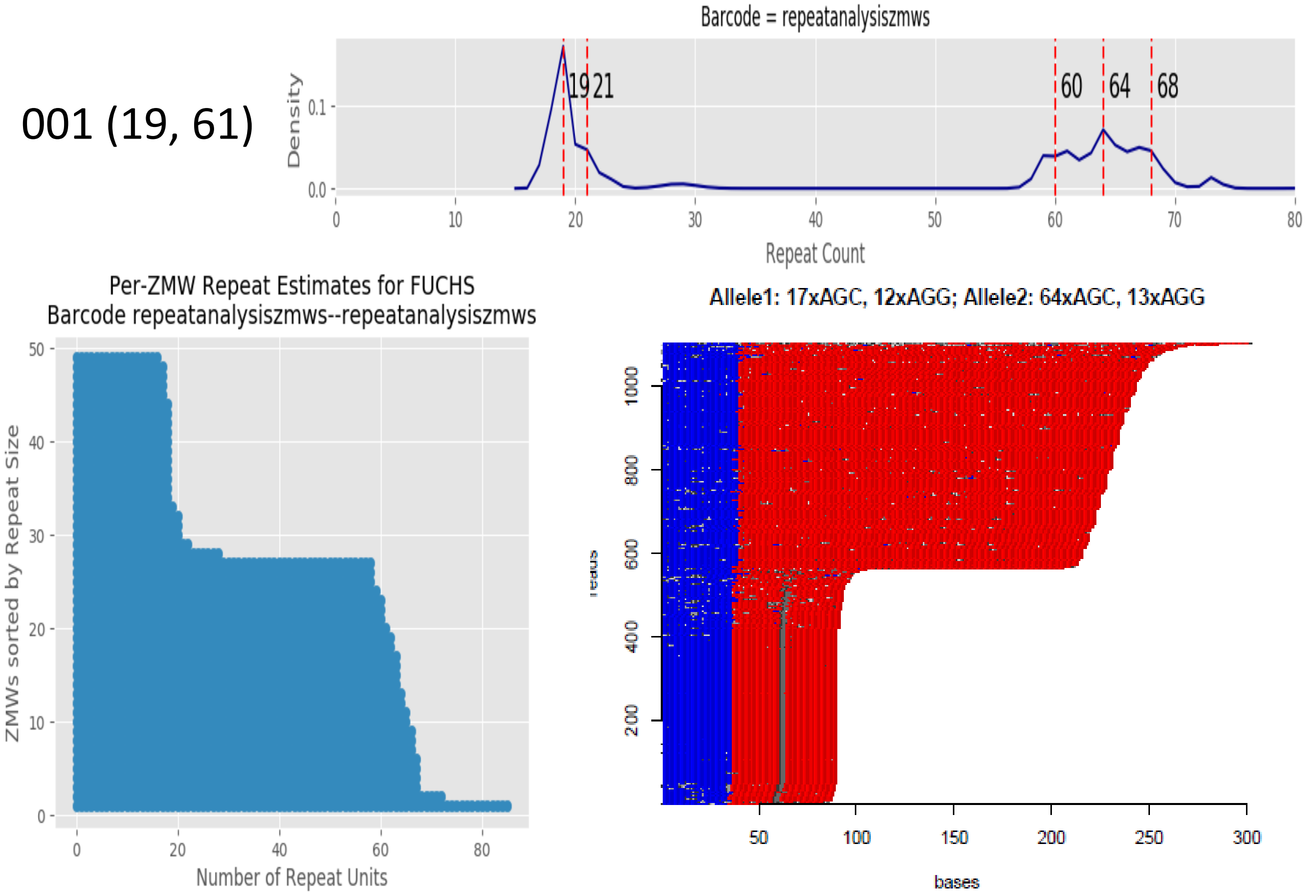

021 (15, 72)

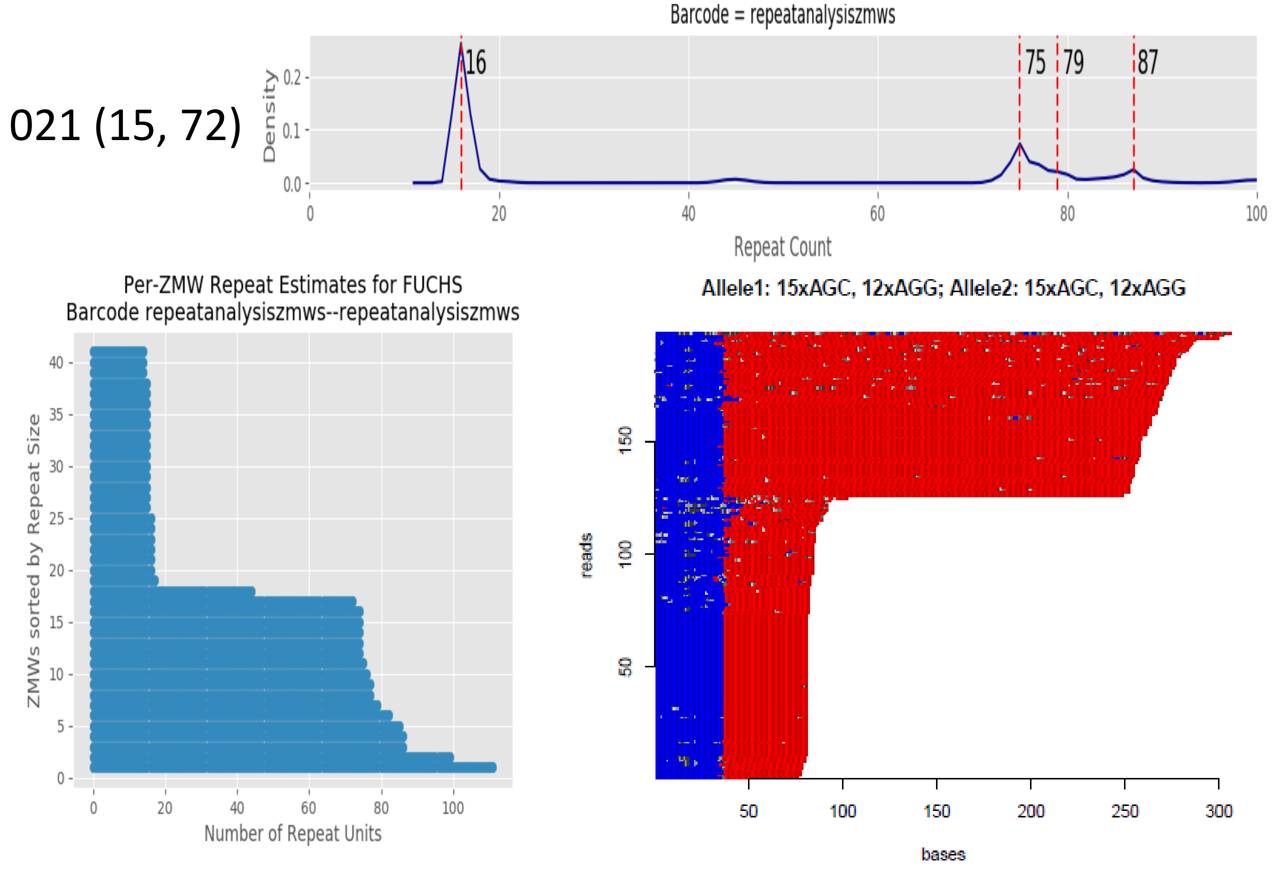

023 (16, 74)

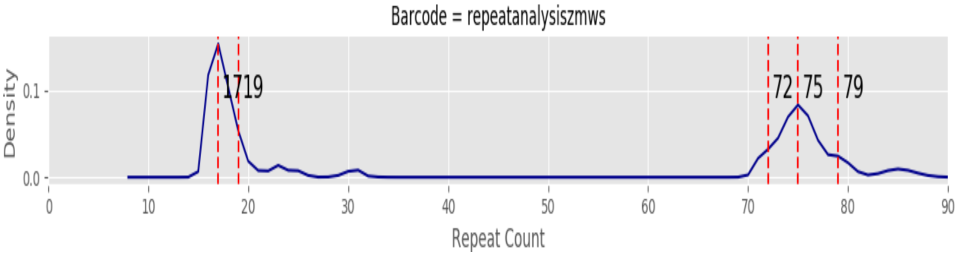

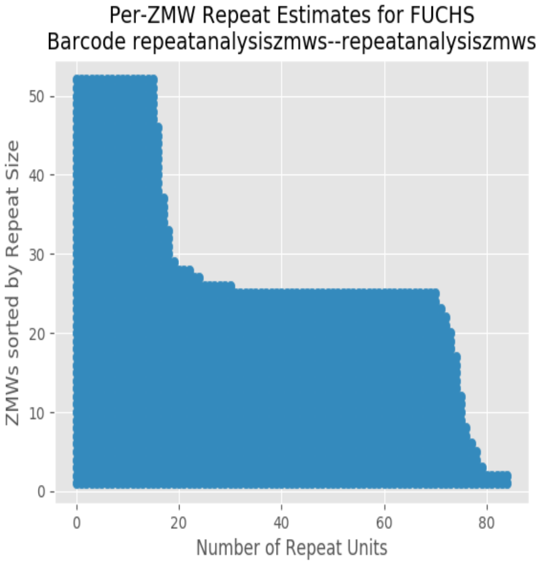

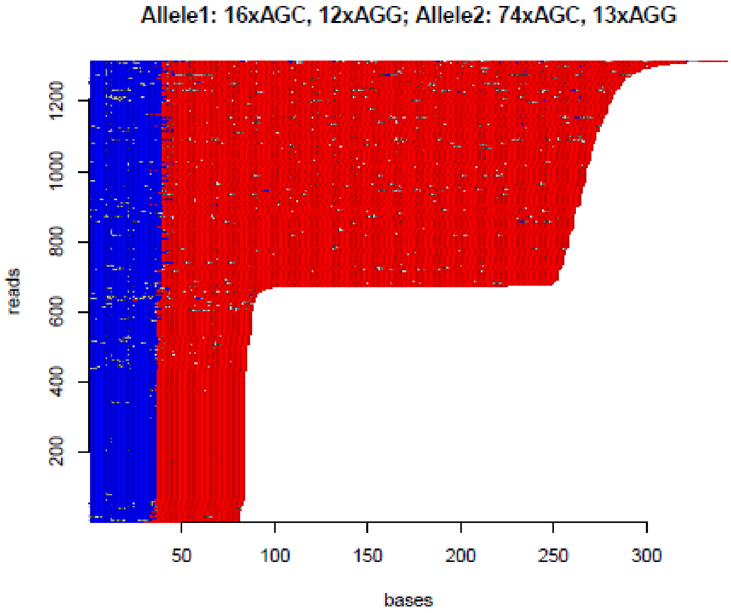

016 (24, 77)

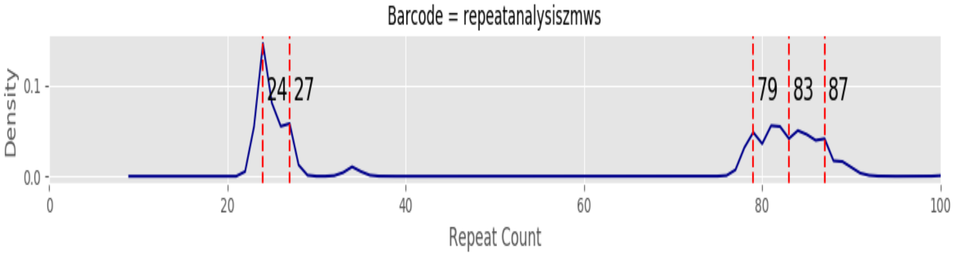

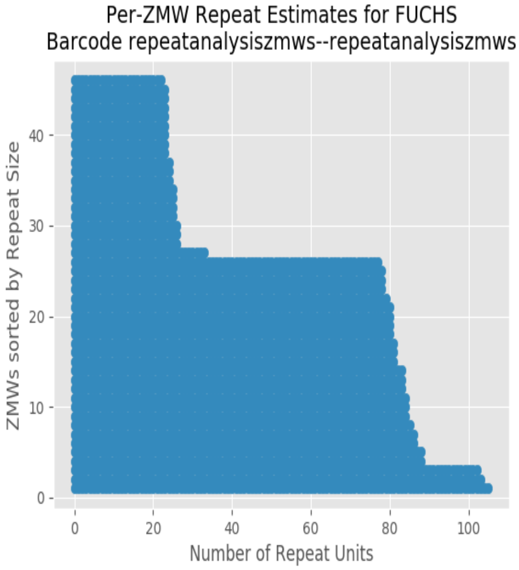

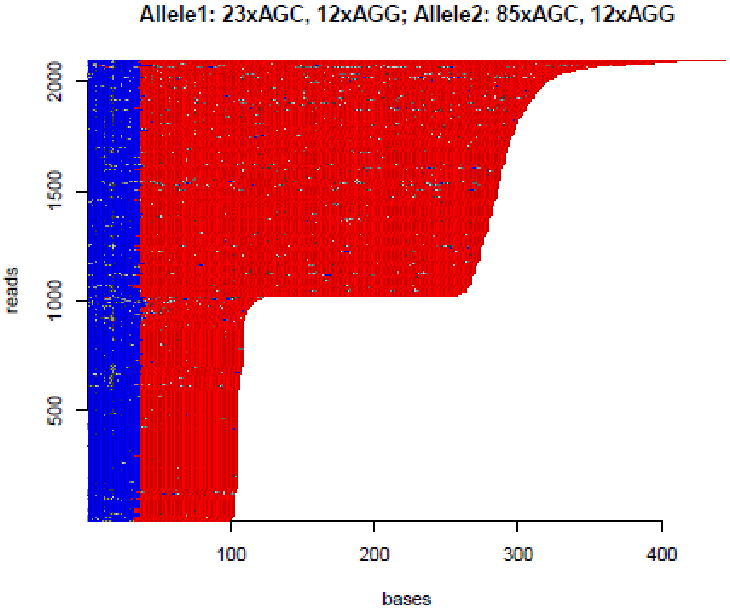

018 (19, 88)

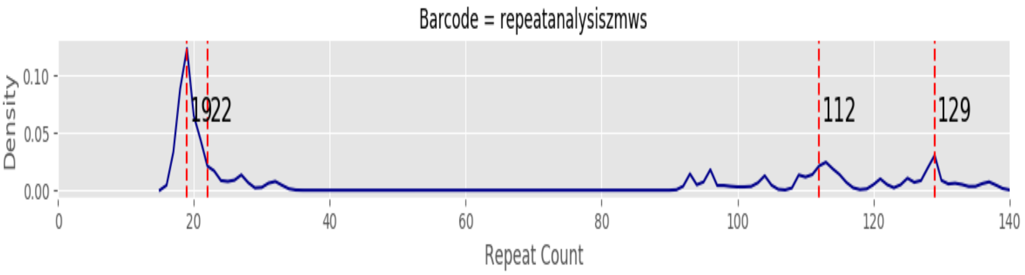

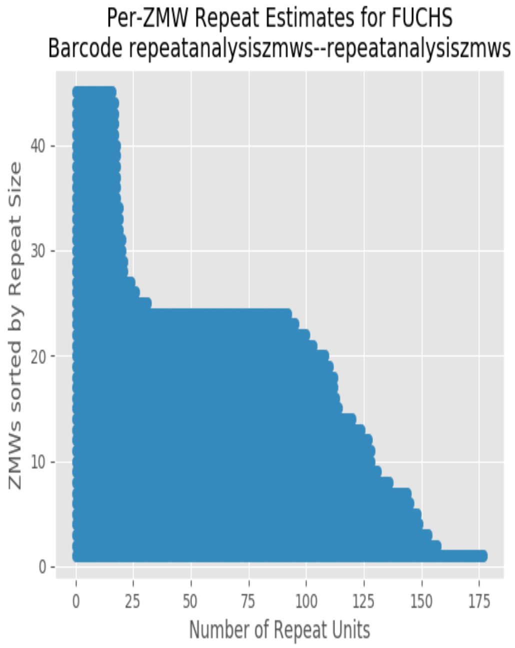

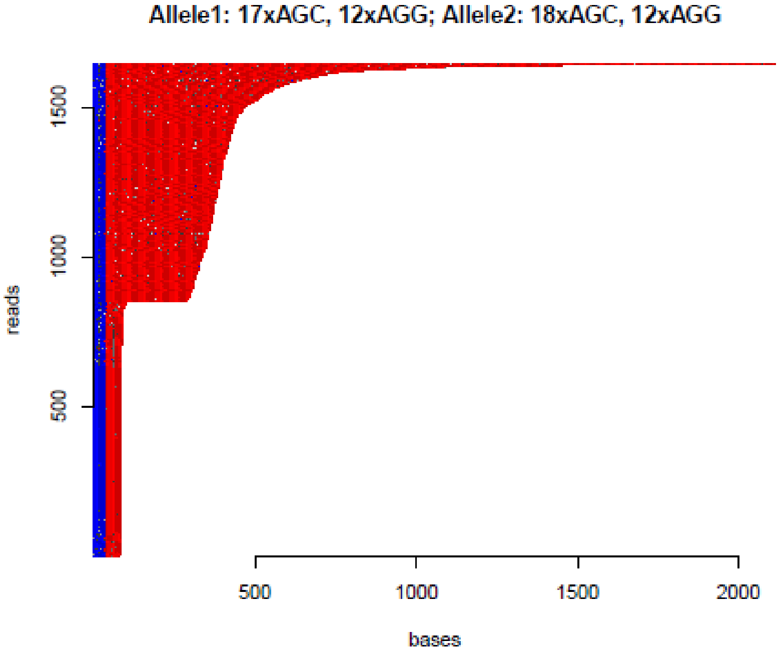

4938 (26, 89)

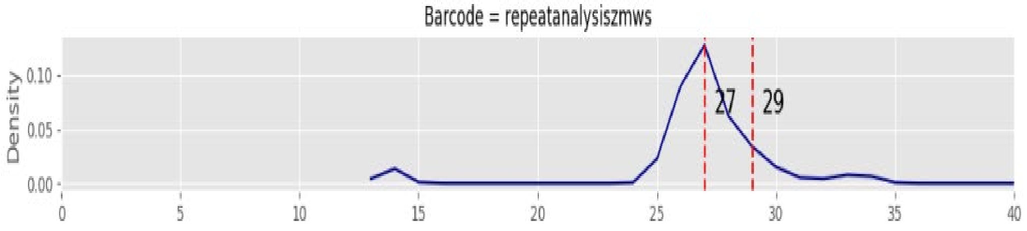

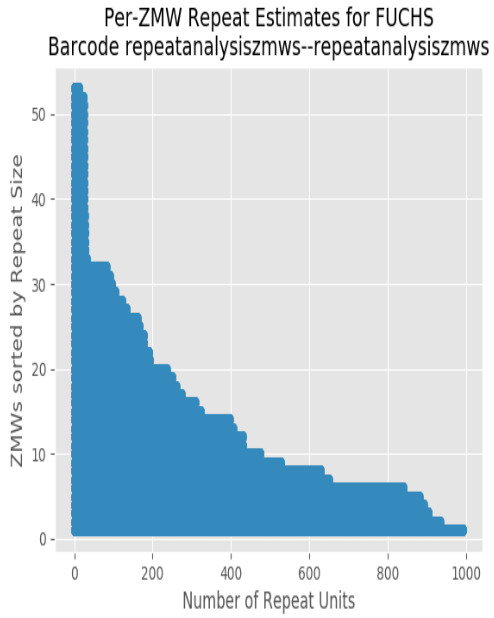

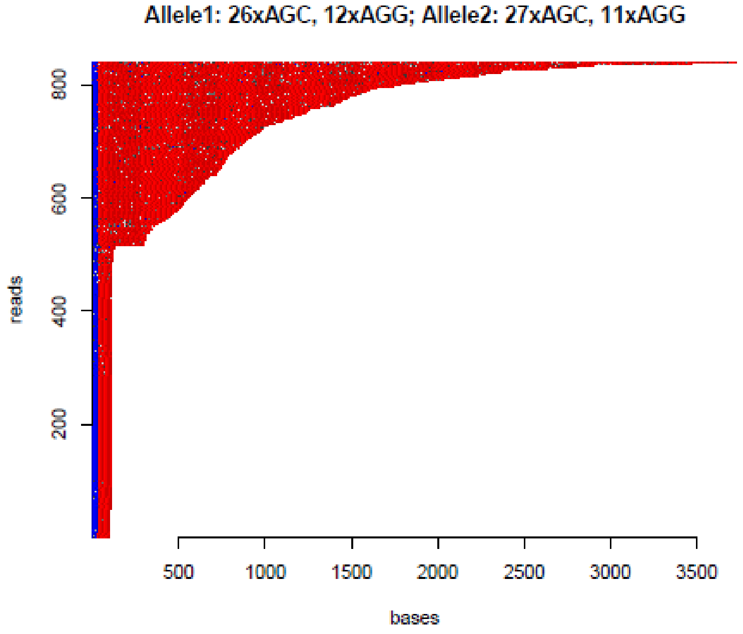

003 (12, 90)

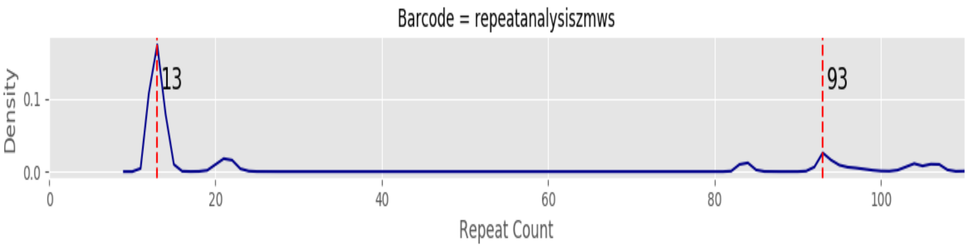

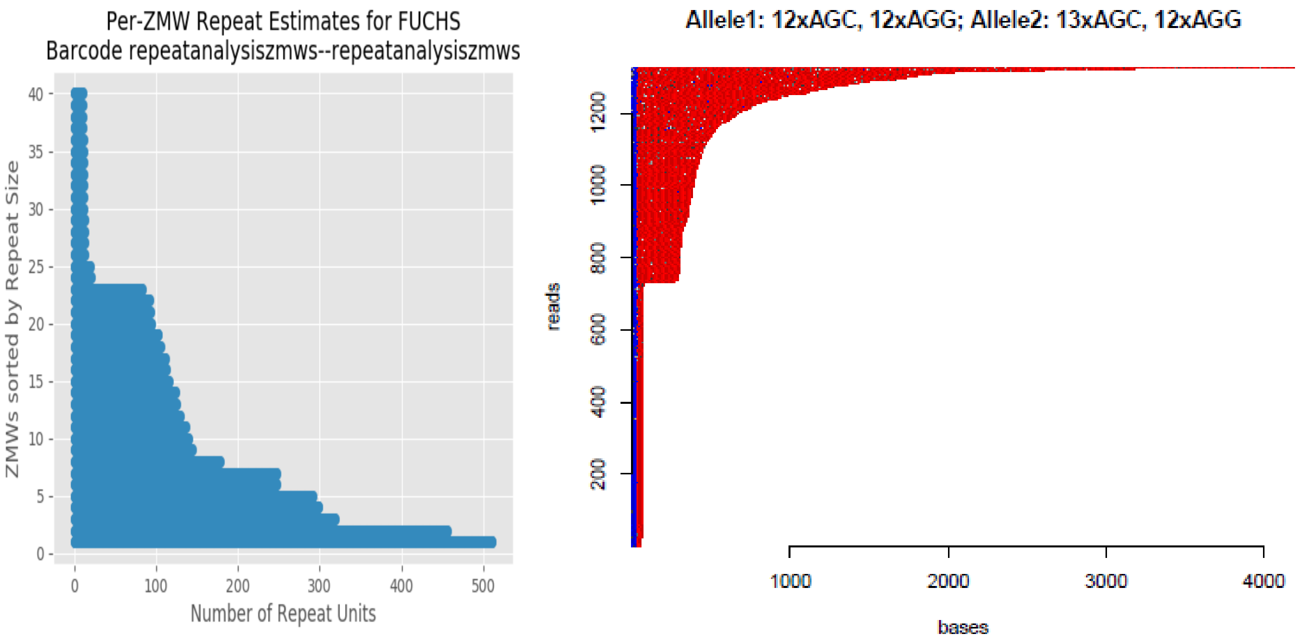

4823 (12, 112)

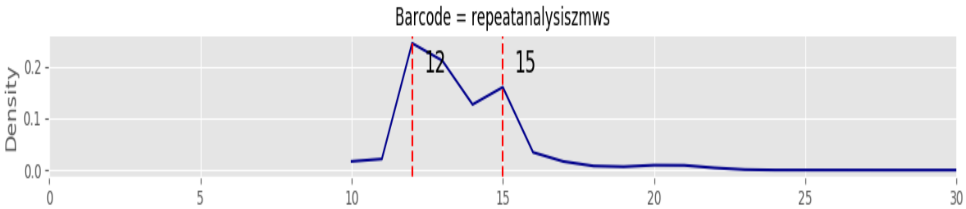

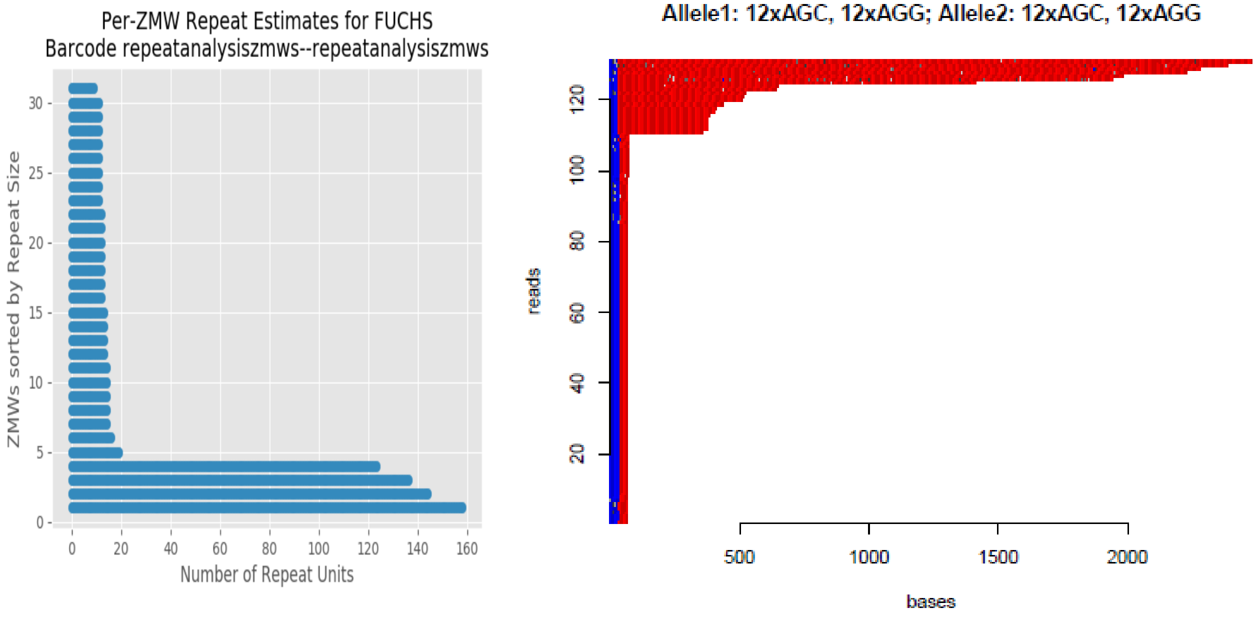

603 (12,15)

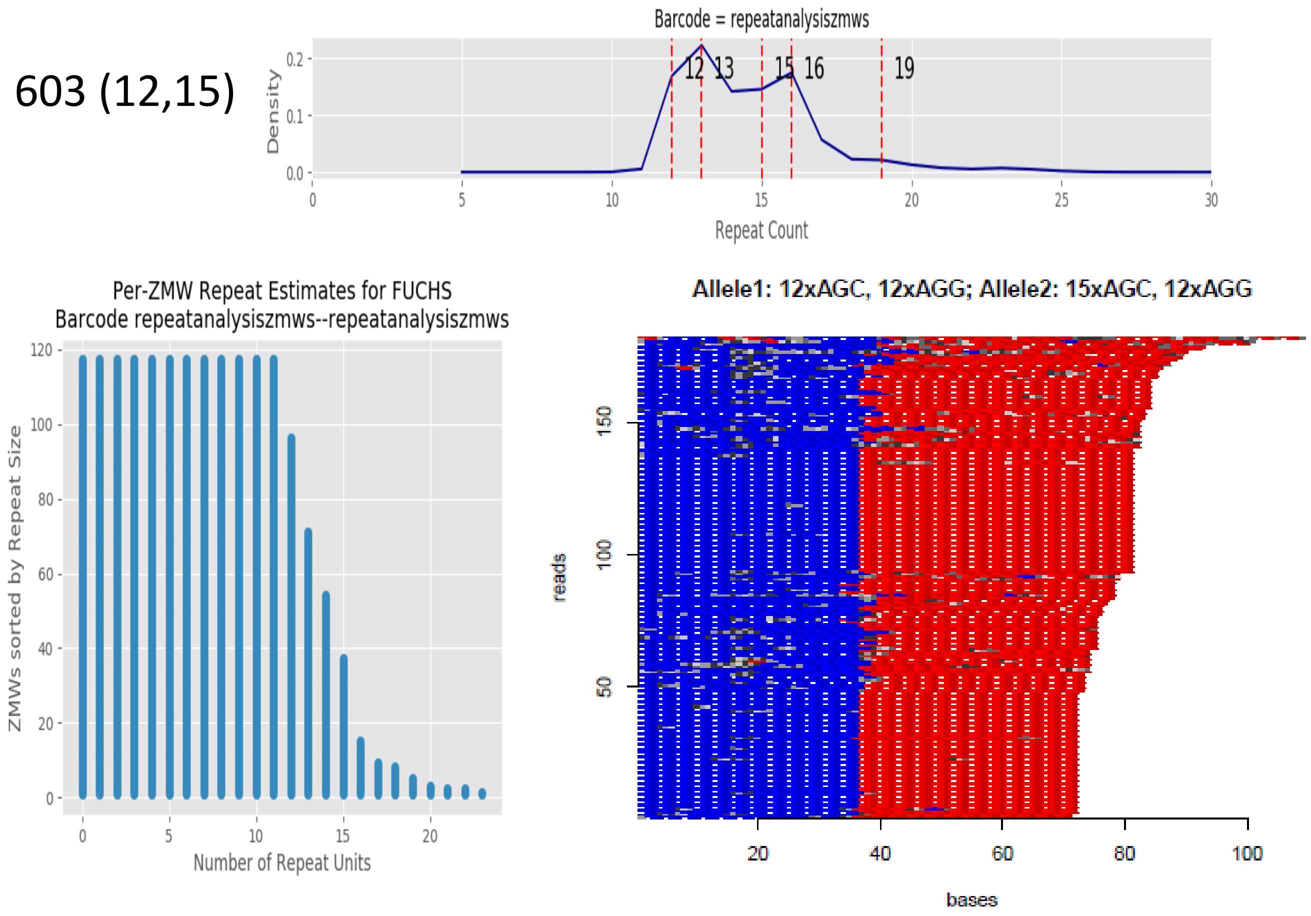

Barcode = repeatanalysiszmws
Density
Repeat Count
12
13
15
16
19
Per-ZMW Repeat Estimates for FUCHS
Barcode repeatanalysiszmws--repeatanalysiszmws
ZMWs sorted by Repeat Size
Number of Repeat Units
Allele1: 12xAGC, 12xAGG; Allele2: 15xAGC, 12xAGG
reads
bases

026 (12, 16)

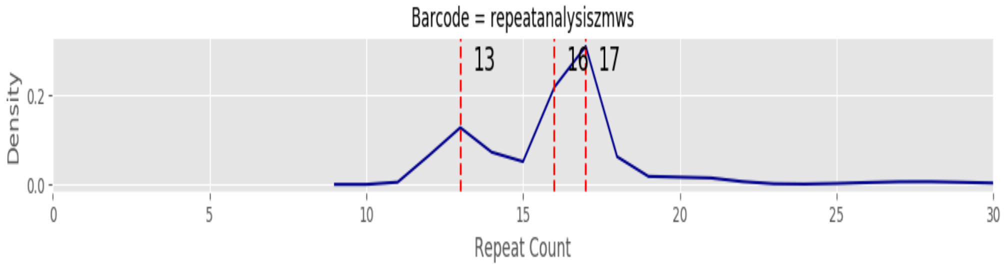

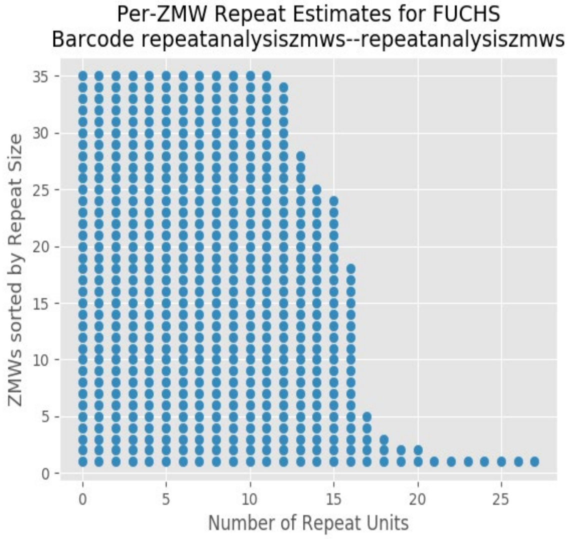

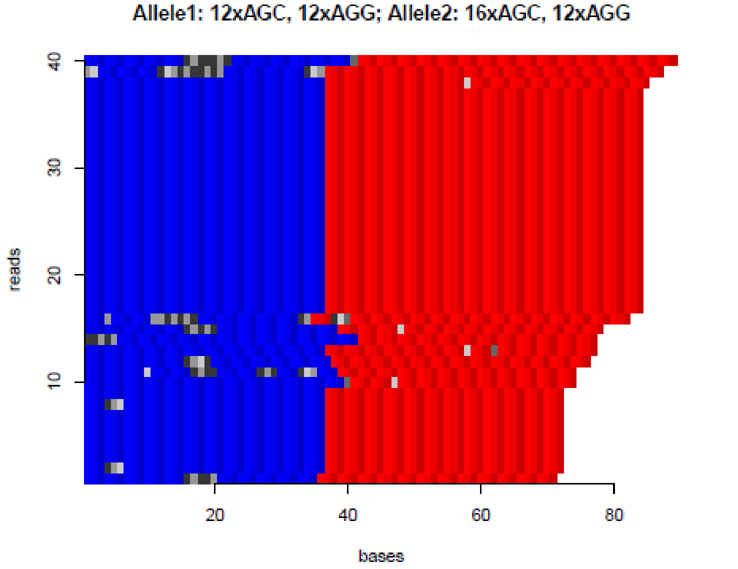

598 (12, 16)

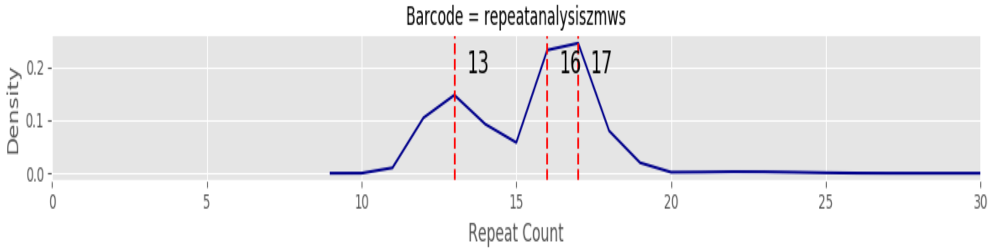

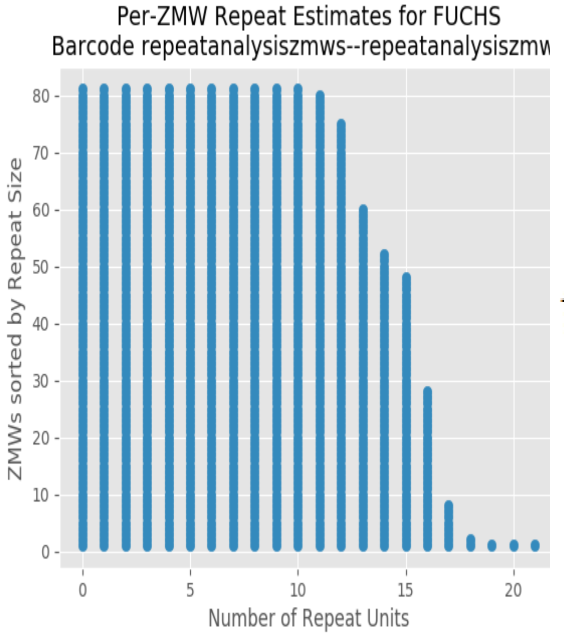

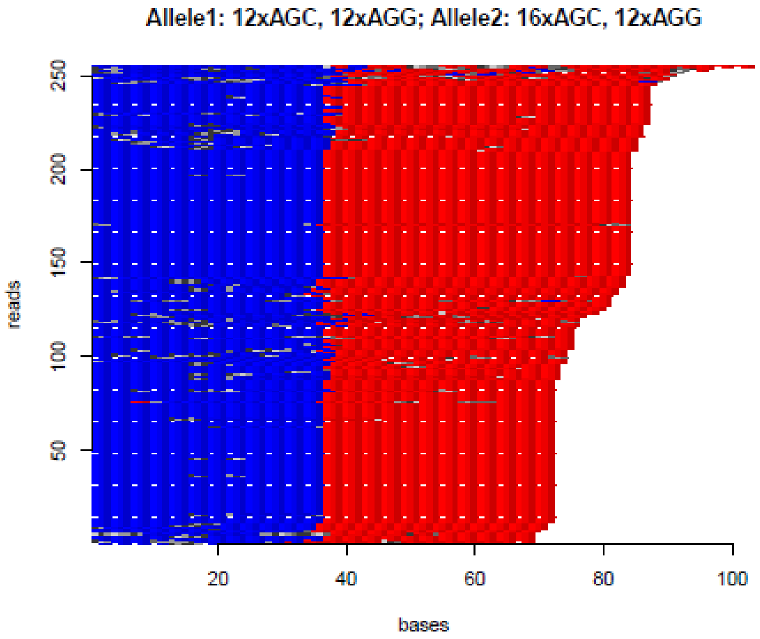

Supplementary Fig. 2. Assessment of repeat length by long-read SMRT sequencing for additional samples. The repeat count histograms, ZMW vs repeat length plots and HTT repeat analysis plots are provided for each sample in this study as described in the legend to Fig. 1. In each case, the STR results for that sample are given in parentheses immediately after the sample name.
